# Adenosine surges: A step forward in understanding antidepressant actions of ketamine

**DOI:** 10.1038/s41380-026-03594-4

**Published:** 2026-04-08

**Authors:** Daniel Dautan, Anderson Camargo, Per Svenningsson

**Affiliations:** 1 Department of Clinical Neuroscience, Karolinska Institutet, Stockholm, Sweden; 2 Department of Basal and Clinical Neuroscience, King’s College London, London, UK

**Keywords:** Neuroscience, Depression

The discovery that a single subanesthetic dose of (R,S)-ketamine induces rapid and long-lasting antidepressant effects by a completely different mechanism represents one of the most substantial breakthroughs in depression pharmacotherapy. The recent study in Nature by Yue and colleagues represents a significant conceptual and technical advance by identifying NMDA-independent transient adenosine surges as a convergent mechanism underlying these actions [1]. For decades, the delayed onset of conventional monoaminergic antidepressants shaped both clinical expectations and mechanistic hypotheses, reinforcing the idea that meaningful antidepressant efficacy necessarily requires weeks of molecular and synaptic remodeling [2]. However, the discovery of (R,S)-ketamine’s instant antidepressant action not only addressed an unmet clinical need but also uncovered fundamental new insights into the neurobiology of depression. The role of purinergic signaling and specific receptors subtypes, including adenosine receptors, have frequently been linked with neuropsychiatric and mood disorders [3, 4]. The prevailing model emphasizes glutamatergic disinhibition as the primary substrate of ketamine’s antidepressant efficacy, in which the blockade of NMDA receptors on inhibitory interneurons transiently reduces GABAergic tone, leading to a burst in glutamate release and increased excitatory drive [5]. This enhanced neuronal excitation promotes brain-derived neurotrophic factor (BDNF) release in the synaptic cleft, which in turn triggers downstream signaling pathways required for the formation, maturation, and function of new synapses [2]. However, in this context, it is noteworthy that disinhibitory effects like ketamine action can also be elicited by the NMDA receptor antagonist memantine without resulting in antidepressant actions [2]. There is, indeed, accumulating evidence demonstrating that ketamine’s molecular targets extend beyond the antagonism of NMDA receptors [2]. Yue and colleagues provide evidence that ketamine and ECT trigger fast surges in extracellular adenosine within the medial prefrontal cortex (PFC). While the work of Yue and colleagues identify the PFC as the primary site, clinical effects likely involves a broader network, such as the hippocampus which has previously been implicated in ketamine-mediated adenosine mechanisms [6].

**Fig. 1:**
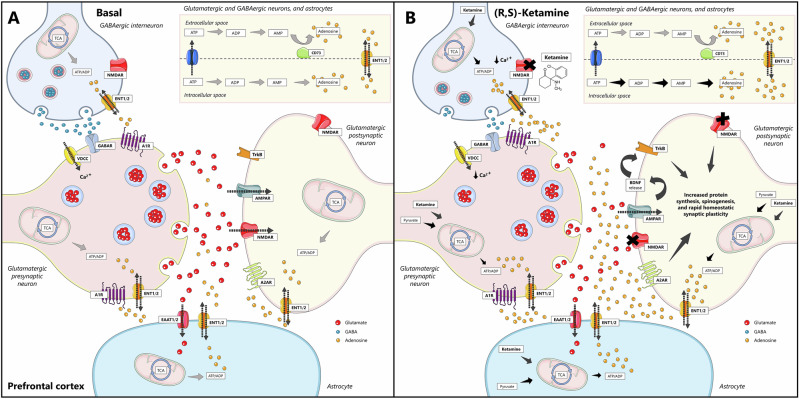
Model summarizing putative mechanisms induced by (R,S)-ketamine-triggered adenosine surges in the medial prefrontal cortex. Schematic showing a cortical synaptic circuit and the effects of glutamate, GABA, and adenosine signaling under basal conditions (**A**) and in response to (R,S)-ketamine (**B**). (R,S)-Ketamine blocks open noncompetitive NMDA receptors on excitatory or inhibitory neurons to impact downstream signaling pathways. The NMDA blockade by (R,S)-ketamine elicits a reduction in Ca2+ levels in GABAergic interneurons, thus resulting in decreased GABA release over excitatory glutamatergic neurons. Concomitantly, independent of NMDA receptors, (R,S)-ketamine reduces ATP/ADP ratios and increases adenosine intracellularly through direct metabolic modulation of pyruvate levels and the tricyclic acid (TCA) cycle. This surge of adenosine is followed by equilibrative nucleoside transporter (ENT1/2)-mediated efflux into the extracellular space. Increased extracellular adenosine levels acts on presynaptic adenosine A1 receptors to reduce Ca+ levels, neuronal activity, and glutamate release. Stimulation of A1 receptors, together with postsynaptic A2A receptors, induces AMPA-BDNF-dependent protein synthesis. This may elicit rapid homeostatic synaptic plasticity, spinogenesis, and antidepressant actions. Abbreviations: A1R — Adenosine A1 Receptor; A2AR — Adenosine A2A Receptor; ADP — Adenosine Diphosphate; AMP — Adenosine Monophosphate; AMPAR — α-Amino-3-hydroxy-5-methyl-4-isoxazolepropionic Acid Receptor; ATP — Adenosine Triphosphate; BDNF — Brain-Derived Neurotrophic Factor; CD73 — ecto-5′-nucleotidase; ENT1/2 — Equilibrative Nucleoside Transporter 1 and 2; GABAR — γ-Aminobutyric Acid Receptor; NMDAR — N-Methyl-D-Aspartate Receptor; TCA — Tricarboxylic Acid cycle; TrkB — Tropomyosin Receptor Kinase B; VDCC — Voltage-Dependent Calcium Channel. Figure created using SMART - Servier Medical Art.

The work by Yue and colleagues is notable not only for its technical sophistication but also for its unifying ambition. By deploying genetically encoded adenosine sensors with high temporal resolution [7], combined with cell-type-specific metabolic and receptor manipulations, the authors provide compelling evidence that ketamine and electroconvulsive therapy (ECT), another rapid-acting antidepressant intervention, trigger fast, dose-dependent, spatially restricted surges in extracellular adenosine within mood-regulatory circuits, particularly the medial prefrontal cortex. Ketamine increased adenosine both in normal mice and in mice subjected to chronic restraint stress. Ketamine increases intracellular adenosine through metabolic modulation of the TCA cycle in mitochondria, followed by equilibrative nucleoside transporter-mediated efflux into the extracellular space (Fig. 1). While the use of sensors offers high temporal and spatial resolution, it remains an open question to which extent these adenosine surges occur within the synaptic cleft and in the extra synaptic space, and how distinct receptor pools coordinate circuit-wide synchronization.

Adenosine is known to increase upon neuronal excitation [8], but ketamine-induced adenosine is reported to occur independent of alterations in neuronal activity. Furthermore, Yue and colleagues report that pharmacological stimulation of adenosine receptors exerts antidepressant action. Likewise, genetic or pharmacological antagonism of adenosine receptor signaling counteracts the antidepressant efficacy of ketamine across multiple behavioral paradigms and depression models. As highlighted by Licinio and Wong [9], the later finding has implications for the consumption of methylxanthines, particularly caffeine, in connection to ketamine and ECT therapies. Indeed, caffeine acts as an adenosine receptor antagonist and could therefore potentially counteract beneficial actions of ketamine and ECT. However, the situation is multifaceted as caffeine per se reduces the risk of depression [10].

The findings by Yue and colleagues reconcile synaptic, metabolic, and circuit-level observations into a coherent, yet incomplete, model in which ketamine transiently shifts cellular energy balance, leading to controlled adenosine release and subsequent adenosine receptor engagement (Fig. 1). These results place adenosinergic signaling at the center of rapid antidepressant actions of ketamine and agree with previous results from our research group published in Molecular Psychiatry [6]. In our study [6], ketamine was shown to rapidly suppress evoked glutamate release through a retrograde adenosinergic feedback mechanism acting at inhibitory presynaptic adenosine A1 receptors (A_1_Rs). Using a combination of in vivo amperometric glutamate recordings with high temporal resolution, synaptosomal preparations, and primary glutamatergic cortical neuronal cultures, we consistently demonstrated that ketamine reduced glutamate release, synaptic vesicle recycling, and presynaptic kinase activity in an A_1_R -dependent manner. Importantly, systemic pharmacological antagonism of A_1_Rs not only reversed these synaptic effects but also counteracted ketamine’s antidepressant-like actions in behavioral assays.

In the study by Yue and colleagues, both A_1_R and A_2A_R exert antidepressant actions and genetic deletion of either A_1_R or A_2A_R counteracted the antidepressant efficacy of ketamine and ECT. It is also interesting to mention that the ability of ketamine to increase BDNF levels is completely abolished in A_1_R and A_2A_R knockout mice, further reinforcing the pivotal role of the adenosinergic system in the mechanisms elicited by ketamine. These data agree not only with the aforementioned pharmacological data but also with previous demonstrations that cortical A_1_R overexpression is associated with antidepressant-like effects, while A_1_R knockout mice displayed depressive-like phenotypes [11]. However, the data of Yue and colleagues are not consistent with the reports that pharmacological or genetic antagonism of A_2A_Rs globally [12] or locally in the lateral septum [13] cause antidepressant actions. However, a closer examination of the results from Yue and colleagues reveals an important asymmetry between A_1_R and A_2A_R receptor subtypes. Selective activation of A_1_Rs was sufficient to reproduce both the acute and sustained antidepressant-like effects of ketamine, whereas A_2A_R activation produced only transient behavioral improvements [1]. This probably relates to the fact that the main anatomical site of adenosine in mediating ketamine actions is in the PFC, a brain region with much higher expression of A_1_R than A_2A_R [8]. Although adenosine acutely suppresses synaptic transmission via A_1_R activity, this reduction in activity may serve as the trigger for a homeostatic synaptic plasticity response, a mechanism now central to models of ketamine’s rapid antidepressant effects [2]. Similar to the NMDAR-at-rest pathway described for ketamine, transient activity suppression can inhibit eEF2K, relieving local translational brakes and enabling a rebound increase in BDNF synthesis [14]. This BDNF increase promotes synaptic potentiation, providing a coherent framework linking acute adenosine signaling to the enduring circuit alterations underlying ketamine’s therapeutic actions.

A_1_R are high-affinity, Gi/o-coupled receptors that exert potent inhibitory control over neurotransmitter release, neuronal firing, and synaptic vesicle dynamics [8]. They are strategically positioned at presynaptic terminals, where they function as sensors of metabolic stress and network activity, providing rapid negative feedback to stabilize circuit function. An interesting aspect of the study by Yue and colleagues is the dissociation of antidepressant efficacy from neuronal hyperactivity. By demonstrating that ketamine reduces intracellular ATP/ADP ratios and increases extracellular adenosine without inducing excessive calcium signaling or seizure-like activity, the authors challenge the assumption that rapid antidepressant responses require heightened excitatory states [2]. This observation is consistent with the inhibitory nature of A_1_R signaling and with earlier findings showing reduced presynaptic glutamate release following ketamine administration [15, 16]. From this perspective, ketamine’s antidepressant action may be better understood as a rapid rebalancing of dysfunctional circuits through metabolically driven inhibitory feedback, rather than only through amplification of excitatory transmission [17]. Previous work on ketamine’s effects on synaptic plasticity has provided evidence that ketamine, in circuit-dependent manners, regulates both Hebbian and homeoplastic synaptic plasticity [18]. It is likely that the adenosine surge mediated by ketamine promotes homeoplastic synaptic plasticity where excitatory synapses are strengthened in response to suppression of glutamate neurotransmission. To further elucidate this topic, it will be critical in future work to simultaneously record extracellular adenosine and glutamate levels along with neuronal activity. While challenging, this could be achieved by state-of-the-art fiber photometry technology.

While these new insights provide a compelling mechanistic framework related to rapid antidepressant actions, they also raise important translational considerations. Yue and colleagues have already initiated the process of developing ketamine derivatives that directly boost intracellular TCA to generate increased adenosine levels [1]. Both deschloroketamine and deschloro-N-ethyl-ketamine induced stronger adenosine surges than ketamine. This occurred independently of NMDA receptor antagonism and resulted in potent antidepressant effects in behavioral tests. Another translational direction is to develop A_1_R stimulatory approaches. A_1_Rs are widely expressed outside the central nervous system, including in the heart, vasculature, kidneys, and immune cells [19]. Peripheral A_1_R activation produces bradycardia, atrioventricular conduction slowing, hypotension, and sedative effects [19]. As a result, systemic pharmacological targeting of A_1_R as an antidepressant strategy carries an inherent risk of systemic side effects that may limit clinical applicability. Strategies that enhance central A_1_R signaling indirectly, by modulating adenosine metabolism, transport, or local release dynamics, may offer a more favorable therapeutic window than direct A_1_R agonists. Alternatively, circuit-targeted delivery approaches or biased agonists that preferentially engage central over peripheral signaling pathways may help mitigate cardiovascular, sedation and autonomic risks [20]. This is the case with the A_1_R agonist that selectively activates brain-enriched Gαob, BnOCPA, which induces analgesia at high doses without cardiorespiratory depression [21]. An alternative approach could be to inhibit adenosine kinase and thereby increase adenosine and indirectly stimulate A_1_Rs [22].

Taken together, by combining state-of-the-art imaging, metabolic analysis, and behavioral paradigms, the recent study by Yue and colleagues provides a compelling and integrative account of how rapid antidepressant efficacy by ketamine can emerge from transient and local neuromodulatory surges of adenosine. This study significantly extends to earlier data identifying A_1_Rs as necessary mediators of ketamine’s synaptic and behavioral effects, thereby consolidating A_1_R signaling as a central mechanistic node (Fig. 1). At the same time, the peripheral expression of A_1_R and the multidimensional nature of depressive symptomatology emphasize the need for precision in applying these insights clinically. Together, these studies move the field closer to a coherent and biologically grounded understanding of rapid antidepressant action, while also delineating the challenges that must be addressed to convert mechanistic clarity into safe, effective, and targeted therapies against depression.
